# Eastern North American Monarch Butterfly Conservation Needs and Opportunities: What the Science Tells Us

**DOI:** 10.3390/insects17030235

**Published:** 2026-02-25

**Authors:** Karen S. Oberhauser

**Affiliations:** Department of Entomology, University of Wisconsin-Madison, Madison, WI 53706, USA; koberhauser@wisc.edu

**Keywords:** monarch butterfly, population drivers, conservation strategies

## Abstract

Monarch butterfly numbers in North America have been declining since the early 2000s. This review focuses on the causes of this decline in the eastern migratory population, found roughly east of the Rocky Mountains, and ways to achieve population sustainability. Drivers of eastern North American monarchs’ decline include (1) the loss of breeding habitat, caused mainly by the loss of their milkweed host plants in agricultural fields after adoption of genetically modified, herbicide tolerant corn and soybeans, (2) weather, particularly warmer and drier conditions that could push monarchs farther north and lead to lower numbers, and (3) increasing insecticide use. Making habitat broadly available to monarchs across their migratory cycle will make them more resilient, and better able to survive weather-driven declines, and even small tracts of land can provide needed habitat. Sites that are isolated from other habitat patches contain higher egg, larva, and adult monarch densities. Good monarch habitat should include a diversity of native milkweed species and nectar plants that will thrive in local growing conditions. Monarch egg and larva density increases with increasing milkweed density and monarch survival is higher when larvae are less crowded, so a good general rule is that more milkweed is better.

## Simple Summary

Monarch butterfly numbers in North America have been declining since the early 2000s. This review focuses on the causes of this decline in the eastern migratory population, found east of the Rocky Mountains, and ways to achieve population sustainability. One key driver of monarchs’ decline is the loss of breeding habitat, caused mainly by the loss of their milkweed host plants in agricultural fields after widespread adoption of genetically modified, herbicide tolerant corn and soybeans and the associated increase in herbicide use. Weather is another driver of monarch population numbers, and climate modeling suggests that warmer and drier conditions in the future could push monarchs farther north or simply lead to lower numbers. The growing use of insecticides to control insect pests has also been implicated in declining monarch numbers. Making habitat broadly available to monarchs will make them more resilient, and better able to survive weather-driven declines. While large tracts of high-quality land will be needed to achieve a sustainable monarch population, even small tracts of land can provide needed habitat. Widespread availability of a diversity of native milkweed species and nectar plants are important components of quality monarch habitat.

## 1. Introduction

### 1.1. Need for Conservation

Monarch butterfly numbers in North America have been declining since the early 2000s, as evidenced by decreased area occupied in their Mexican overwintering sites. The 2014 petition to list monarchs as threatened under the Endangered Species Act (ESA) and subsequent 2024 recommendation by the U.S. Fish and Wildlife Service (USFWS) that monarchs receive formal protection under the ESA [1] provides recent impetus to monarch conservation action. However, interest in monarch conservation predates the USFWS decision by decades, probably stemming from the discovery in 1975 [2] that most monarchs from the eastern half of the United States and southeastern Canada travel thousands of miles to a relatively small area in central Mexico during a spectacular fall migration. Early conservation focus was on these overwintering sites, but degradation from logging and other factors slowed drastically after establishment of the Monarch Butterfly Biosphere Reserve in Mexico [3,4,5].

While monarchs have a world-wide distribution, migratory populations in North America (the eastern and western migratory populations) comprise the vast majority of monarchs in the world. Of these two populations, the much larger eastern population is the focus of this article. A recent review [6] summarizes population trends and their drivers for the western population. The eastern population is found roughly east of the Rocky Mountains, extending into southern Canada and northern Mexico. Monarchs are also found in South America, Central America and the Caribbean; many Pacific Islands including Australia and New Zealand, and Hawaii; the Iberian Peninsula; mid-Atlantic Islands; and the Indian Ocean [7,8], but genetic evidence suggests that monarchs originated in North America [9,10,11].

### 1.2. Annual Migratory Cycle

**Figure 1:**
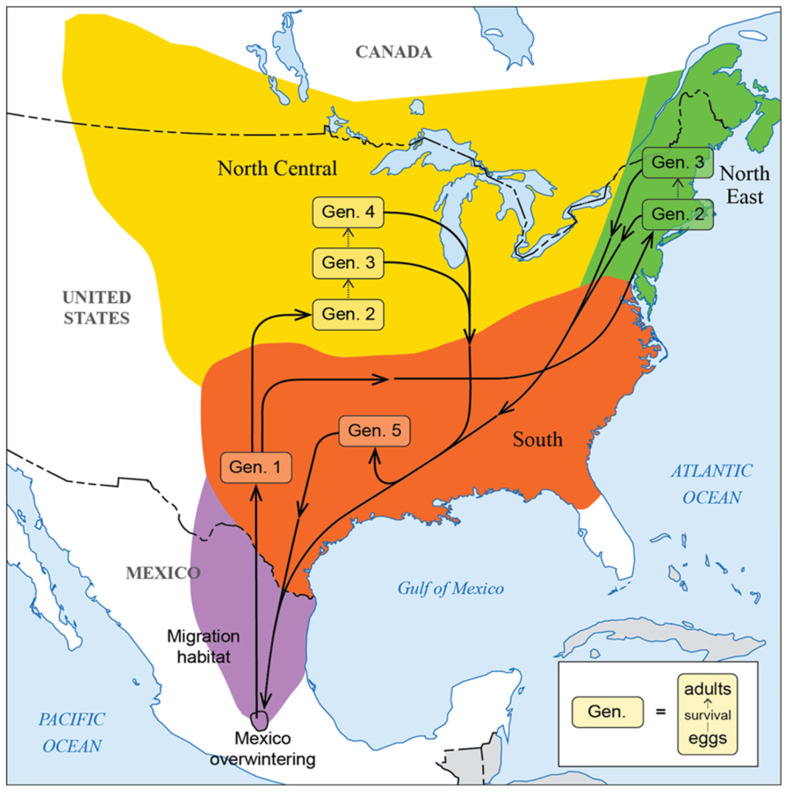
Annual monarch migration in Eastern North America. Colors refer to regions where different generations occur, and each line represents either movement from one region to another, new generations, or (within boxes) survival from one stage to the next. From [13].

The monarch migratory cycle includes (1) breeding for multiple generations across a wide geographic range and diverse ecological conditions, (2) migrating south, (3) overwintering in an area orders of magnitude smaller than the breeding and migratory ranges, and (4) migrating north (Figure 1). Each part of this migratory cycle has ecological and biological requirements that require conservation focus. Monarchs are well-studied by professional scientists aided by thousands of “citizen scientists”, members of the public who volunteer their time to collect detailed data on monarch breeding, migration, population size, disease and natural enemies, and habitat requirements across the entire annual cycle [12].

*Summer Breeding.* During summer (roughly May through September), three generations of breeding monarchs in the eastern migratory population cover a large area (the green and yellow areas on Figure 1) in the U.S. and southeastern Canada. These non-migratory individuals live from two to six weeks as adults, mating and laying eggs for all but the first few days of their adult life [14]. Female monarchs mate multiple times and probably lay 400–600 eggs during their lifetime [15], although lifetime fecundity is difficult to study in the wild. They lay eggs on most of the over 100 milkweed species found in North America, and their larvae consume leaves and other parts of these milkweed host plants. They gain some protection from predators by sequestering toxic cardenolides from milkweed [16]; levels of cardiac glycosides vary among milkweed species (e.g., [17]).

During their summer breeding period, monarchs require intact habitat with milkweed for egg-laying and larval food, and nectar for adults. In addition to these biotic requirements, summer monarchs are affected by weather, both directly and indirectly. Survival and lifespan are directly affected by temperature extremes, with both very cold and very warm temperatures slowing development and killing monarchs in all life stages [18]. Additionally, temperature extremes and drought can kill the above-ground portion of perennial plants or entire annual plants and decrease nectar production, thus indirectly affecting monarchs by decreasing the availability of food resources.

*Autumn migration.* Individuals in the final generation of each year leave their summer breeding grounds and migrate south and southwest, funneling through Texas and northern Mexico toward wintering sites in central Mexico. These individuals emerge as butterflies beginning in mid-August and continuing through most of September and October, and even early November. Responding to environmental conditions encountered during development and possibly early adult life, especially decreasing daylength (but also day-night temperature differences and host-plant conditions [19], migratory monarchs put reproductive development on hold and enter a state called reproductive diapause, or reproductive dormancy. During the fall migration, monarchs require nectar plants to fuel their migration and overwintering period. They only migrate during the day, and thus require safe places to stop at night, usually in trees. Monarchs synthesize lipids from sugars in plant nectar as they migrate south [20,21,22]; these lipids are gradually depleted over the course of the winter [23].

**Figure 2:**
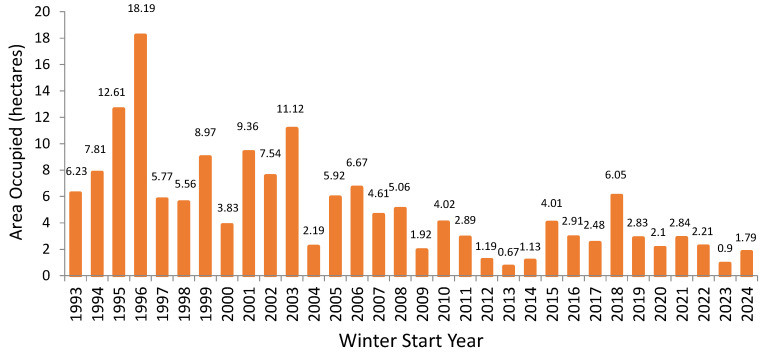
Area occupied by monarchs in the monarch overwintering sites in the Trans-Mexican Volcanic Belt. Data from 1993–2003: Monarch Butterfly Biosphere Reserve (MBBR, CONANP). Data from 2003–2025: WWF-Telcel Alliance, in coordination with the MBBR.

*Overwintering in Mexico.* From early November through approximately mid-March, monarchs overwinter in the mountains of central Mexico. While the actual area containing monarchs is small (up to 14 colonies cover an area that has ranged from about 20 to under one hectare in the past 30 years), the Mexican government has protected 56,259 hectares as the Monarch Butterfly Biosphere Reserve in the Trans-Mexican Volcanic Belt. This UNESCO World Heritage site provides a forested buffer to the overwintering locations. The area covered by butterfly colonies has been measured completely since the early 1990s (see Figure 2), with less complete monitoring occurring before that [24]. Because most monarchs in the eastern migratory population spend the winter in these sites, this area is assumed to reflect the size of the population (e.g., [25,26,27]).

*Spring migration.* Monarchs leave their Mexican overwintering sites beginning in early- to mid-March and fly northward, mating and laying eggs along the way. These migrants, who started their lives in the northern breeding range the previous fall, recolonize roughly the southeastern quarter of the U.S. (the orange area on Figure 1) before dying after an adult lifespan of about eight to nine months. Their offspring, the first generation of the new year, recolonize the northern breeding range as milkweed becomes available, and the cycle continues [28,29].

*Exceptions.* While the vast majority of monarchs in the eastern migratory population follow the pattern described above, some individuals leave their northern breeding grounds early and undergo what is sometimes called a “pre-migration migration”. These individuals are not in reproductive diapause, and mate and lay eggs as they move south [30]. Most of their offspring probably join the fall migration.

Other individuals leave during the main migratory wave but break diapause in the south and breed along the Gulf Coast and farther inland, or on islands in the Gulf of Mexico [30,31,32,33,34,35]. Because breeding in these locations is ongoing throughout the winter, offspring of winter breeders probably do not migrate to the Mexico wintering sites but produce additional non-migratory generations. Winter breeding individuals have high rates of infection with *Ophryocystis elektroscirrha* (OE), a protozoan parasite with severe fitness consequences that infects monarchs and their close relatives [36,37]. They are also likely to be found on the non-native milkweed *Asclepias curassavica* (tropical milkweed) found in cultivated gardens as a host plant (e.g., [30,33,34]). Because native milkweeds die back in the fall and winter except in very wet years, it appears that cultivated tropical milkweed encourages fall and winter breeding. Additionally, it can lead to increased transmission of OE because multiple generations of monarchs utilize the same host plants, allowing build-up of OE spores on the plants. Monarchs returning from the Mexican wintering sites can interbreed with these individuals, and the disease can be transmitted to the offspring of a healthy individual that mates with an infected individual [38]. While offspring of winter breeders might join the northward migration in the spring, high levels of OE are likely to minimize positive impacts on the overall population.

Other exceptions to the general pattern represented in Figure 1 include the movement of individuals between the eastern and western migratory populations. Tagging studies have documented movement of some individuals from the western U.S. to Mexican wintering sites [39,40], and it is likely that movement from the Mexican wintering sites to the western U.S. occurs as well [41,42]. This interchange has probably caused a lack of genetic differentiation between the two populations [43,44], but the degree to which one population can replenish the other is unknown. Finally, some monarchs overwinter without breeding in the southeastern U.S. and along the gulf Coast [45,46,47].

## 2. A Species in Decline

Long-term data bases (summarized in [12]) document population trends during all stages of the monarch annual cycle and allow population and climate modeling to project what the future might hold for monarchs. World Wildlife Fund-Mexico and Monarch Butterfly Biosphere Reserve personnel began collecting systematic data on the area covered by overwintering monarchs in 1993 [24]. The spring migration is tracked by Journey North volunteers [48]. Data on summer numbers of adult monarchs are provided by counts North American Butterfly Association (NABA) counts [49] and Butterfly Monitoring Networks in several states, with the longest time series coming from volunteers in IL, OH, IA and MI [50], and a more recent dataset provided by the Integrated Monarch Monitoring Project (IMMP, [51]) beginning in 2016. Data on monarch egg and larva abundance are collected by Monarch Larva Monitoring Project volunteers [52] and the IMMP. Autumn migration studies include censuses on peninsulas that funnel monarchs as they fly south [53], studies of tagged butterflies [54,55], and reports of migratory roost sites to Journey North [56].

In 2023, Shirey and Ries [57] published a review of long-term studies that looked for trends in monarch numbers and attempted to tease apart the factors that were driving these trends. They included 23 studies with ten or more years of data, and divided these studies based on where and when monarchs were measured. All analyses (10) of overwintering area showed a decline (see Figure 2). Whether monarch numbers continued to decline or have leveled off in the past decade is a matter of ongoing debate [58,59,60,61], but it is generally agreed that the current numbers are not high enough to ensure population sustainability [26,58,60,61,62].

Population analyses during the breeding and migratory portions of the annual cycle are more complicated because monarchs cover such a broad area during this time and because the population grows over the course of the summer. Not unexpectedly, these studies are less conclusive. One fall migration study that ended in 2010 showed an increase in monarch numbers over time [63]. Three breeding season studies using adult counts and ending in 2015 found no evidence of a decline over time [64,65,66], but more recent studies using longer time periods do document regional declines in monarch numbers in the Midwestern and Northeastern U.S. [67,68]. Crossley et al. [68] suggested that declines in the Midwest and Northeast may be offset by increases in other regions, but these regions do not include areas that produce large numbers of monarchs (see Other Perspectives on Monarch Status). Two studies of eggs and larvae ending in 2014 found declining egg densities [69] and survival [70].

The difference between the clear trends at the wintering sites and less clear trends during the breeding and migratory season could be caused by biology, if the winter declines are driven by mortality during fall migration and numbers of summer monarchs really are not changing. Alternatively, the difference could be due to sampling issues. If monarchs are being crowded into smaller areas as habitat is lost, surveys could give the impression of constant or even increasing numbers even if the population is declining [71]. The hypothesis that declining monarch numbers at the wintering sites are caused by increased mortality during the migration is not supported by tagging data [55]; if mortality during the fall migration was increasing and thus driving the decline in overwintering numbers, the proportion of tagged monarchs that are recovered in Mexico should be declining. This is not the case. However, a recent analysis of the size of monarch roosts reported by Journey North volunteers during the fall migration documented a decline in the size of these roosts over time, and this decline was more pronounced in more southerly roosts [72]. The overall decline in roost size could be explained by a shrinking population, but the relatively larger decline farther south suggests increased mortality during the migration. Ongoing analyses of these important citizen science data sets (Monarch Watch tagging and Journey North roost observations) may help to clarify the relative roles of decreased breeding and migratory success.

Comprehensive analyses documenting a strong correlation between the size of the eastern migratory population at the end of the summer and the size of the wintering colonies [67,73] provide additional evidence that declines in the number of monarchs wintering in Mexico are driven by decreasing production during the breeding season. Of course, migration and overwintering are also crucial parts of a complex annual cycle, and the relative importance of breeding, migratory, and overwintering conservation practices probably vary from year to year. Thus practices that support each stage of the cycle need to continue [13].

Based on evidence that monarch numbers are declining, in 2014 the Xerces Society for Invertebrate Conservation, the Center for Biological Diversity, the Center for Food Safety, and Dr. Lincoln Brower submitted a petition to the USFWS to list the monarch as a threatened species and give it protection under the Endangered Species Act (ESA). In December 2020, the USFWS announced their determination that the monarch listing was warranted but precluded because other species at greater risk were prioritized. In December 2024 the USFWS announced that monarchs are threatened and should thus receive legal protection under the ESA. They determined that monarchs are likely to become endangered in the foreseeable future through a significant portion of their range [61]. This decision is based on the ongoing decline in monarch populations and the fact that they have not achieved a sustainable population size [26,60]. In late 2025, the USFWS moved the decision on monarch status into the long-term action category, meaning that a ruling is not expected within the next twelve months. The Monarch Joint Venture provides a detailed summary of this decade-long process [74].

The International Union for the Conservation of Nature (IUCN) assessed the status of migratory monarchs and listed them as Vulnerable in 2023 [75]. Meehan and Crossley [59] criticized the IUCN estimate of how monarch numbers were changing over the most recent ten years and argued that a model that incorporated variable change rates should have been used. Their model included a negative density dependent function, which assumes that populations rebound quickly after years of low population size. Crone et al. [76] criticized this approach (see also [60]). While Crone et al. [76] acknowledged that Meehan and Crossley [59] provided one of several reasonable approaches to analyzing population abundance, it was not appropriate to fit a standard density-dependent to a population that is measured every four generations and pooled from such a large area, or to a time series of population sizes as variable as found in monarchs. Additionally, Crone et al. [76] and Thogmartin [60] argue that there is no evidence that monarchs will be able to consistently recover from low population sizes.

## 3. What Is Causing the Decline?

Monarch populations levels are determined by factors that affect recruitment (the number of eggs that are laid and that survive to adulthood) and adult survival and successful navigation of the annual cycle. Recruitment and survival are affected by the availability of quality habitat, weather conditions, and the prevalence of lethal agents (natural enemies such as predators, diseases, parasitoids, and parasites; environmental toxins and pesticides; and other agents of mortality that are often difficult to quantify, such as vehicles).

### 3.1. Habitat Loss

Many factors have affected monarch habitat availability over the past century, and their relative importance is probably changing. Forested area in the eastern U.S. was cleared for farmland and cities in the 1800s and early 1900s, but much of that farmland has since been taken out of cultivation and forests are recovering. North American settlers from Europe converted prairies in the Central Plains to farmland, but early farms in the eastern and central U.S. tended to have small fields with milkweed and nectar plants growing on the edges, and chemical pesticides were not available. Even with the growing predominance of large, cultivated fields after the mid-twentieth century, most weed control methods did not kill milkweed; on the contrary, most milkweed in the Upper Midwestern U.S. in the 1980s and 1990s was found in corn and soybean fields [77]. Thus, early farming practices probably benefited monarchs; in the east, farmland replaced forests that were not good monarch habitat, and the land disturbance that accompanies agriculture promoted the growth of what became eastern monarchs’ most important food source, common milkweed (*Asclepias syriaca*). Because these changes occurred before monarch populations were being measured, it is difficult to assess their impacts. Anecdotal reports of monarch numbers during the fall migration beginning in 1875 indicate a great deal of variation [45] but document no long-term trends.

There is widespread scientific support for concluding that the main cause of monarchs’ population decline is the loss of breeding habitat in the Upper Midwestern U.S. that resulted from the adoption of genetically modified, herbicide tolerant row crops. Beginning in the late 20th century these crops (first Roundup^®^ Ready (Monsanto/Bayer) crops, which are resistant to glyphosate, and then a series of crops resistant to other herbicides) allowed farmers to spray their fields with herbicides after the crop plants emerged, and milkweed quickly disappeared from corn and soybean fields as adoption rates of herbicide tolerant crops went from 7% for soybeans and 0% for corn in 1996 to over 90% in both corn and soybeans by 2014 [78,79,80,81,82]. Several studies have documented a link between monarch declines and herbicide application on agricultural fields, for numbers of overwintering [81,83,84] and summer breeding [66,67] monarchs. Milkweed continues to be lost on the breeding landscape through the conversion of grasslands to cropland [85,86].

The rapid adoption of herbicide tolerant crops in the agricultural fields that supported so many monarchs led to a complex data series. Before the late 1990’s, monarch numbers fluctuated up and down, probably driven by year-to-year weather variation. This period was followed by a period of decline in the first decade of the 21st century, as milkweed disappeared from corn and soybean fields. After this milkweed was gone, monarchs entered a new period of fluctuation around a lower average (Figure 2).

Some researchers have questioned the importance of breeding habitat availability as a driver of monarch numbers [65,87,88], arguing that drivers during migration could be more important. However, ongoing evidence for links between monarch population size and herbicide application [66,67,81,83,84] and a clear mechanistic link between herbicide application and loss of milkweed that had been heavily used by monarchs [77,84,89,90] suggest that breeding habitat loss was a key driver in the steep decline in monarch numbers that occurred in the early twenty-first century.

There has also been habitat loss and degradation in the Mexican wintering sites [3,91,92], decreasing the area available for wintering roosts, but perhaps more importantly resulting in declining microclimatic suitability in the remaining forest [93]. Thogmartin et al. [81] documented a relationship between habitat loss in these sites and monarch numbers, but this effect was much smaller than that caused by breeding habitat loss. Wintering habitat loss has been dramatically curtailed since 2009 [4,94].

### 3.2. Weather

In 1995, Brower [45] suggested that weather is a major driver of monarch numbers, especially winter storms in Mexico (which have killed up to 80% of the population, [95]); cold, wet springs; and dry hot summers. However, detailed and long-term data on the size of the population were not available to test his assertions, and it took 20 years to accumulate enough data from the monitoring programs described above to do so.

We now have documentation of the important impacts of weather on monarchs throughout their annual cycle from both field and lab studies. Cold temperatures cause adult mortality in the winter, especially when combined with precipitation [93,96]. Low temperatures during the spring and summer breeding season can cause larval mortality [18] and slower development [18,97]. Hot temperatures during the breeding season result in shorter lifespans and fewer eggs [15], egg and larval mortality [18,98,99], and smaller individuals [18].

Researchers have used ecological niche modeling to determine that the locations used by monarchs in the winter [100] and summer [101] have specific climatic (temperature and precipitation) conditions. It is likely that hot conditions in the southern U.S. drive monarchs northward in the spring [28,101,102]. Similarly, the ranges of important monarch predators [103] and milkweed [104] are determined by climate. Weather is also likely to affect monarch and host plant phenology, influencing the number and timing of monarch generations and resource availability, although this has not been well-studied.

Given the known impacts of temperature and precipitation on monarchs, it is not surprising that monarch numbers are strongly associated with weather from year to year. Thogmartin et al. [81] showed that, while the most important driver of winter numbers was the amount of glyphosate used in the breeding grounds, weather was also a driver, with hot conditions in the north central U.S., particularly in August, leading to lower monarch numbers. Spring climate in the southern U.S. is also an important driver, with both hot and dry conditions and cold and wet conditions leading to fewer monarchs in the north [66,67,105], as predicted by Brower [45]. While drought conditions during the fall migration can result in lower nectar availability and monarch lipid levels [21], monarchs appear to be able to make up for lower nectar availability during one part of migration as they move southward [21,106].

Associations between weather and monarch numbers could be the result of direct impacts on monarchs themselves or through indirect impacts mediated by the effects of weather on host plant quality and quantity, and nectar availability, all of which affect survival and fecundity [107,108]. It is likely that both indirect and direct effects are important.

Research teams [67,105,109,110] have looked at the relationship between local weather conditions and monarch numbers in the Upper Midwestern U.S. (primarily Oho and Illinois). These detailed analyses of summer numbers documented nuanced interactions. In cooler regions (in the northern part of the breeding range), warmer conditions than normal led to more monarchs, while in warmer regions (the southern part of the breeding rage), cooler conditions led to more monarchs [109,110]. Crossley et al. [68] also found that higher temperatures led to more monarchs in the northern part of the range and fewer in the south.

### 3.3. Insecticides

The use of insecticides to control agricultural and nuisance pests, as well as insects that vector human pathogens, has increased in the past few decades [111]. Many studies show that widely-used insecticides, including organophosphates, pyrethroids, Bt, and neonicotinoids, kill monarchs at concentrations used in the field [112,113,114,115,116,117], although others have shown minimal effects of field-level doses (e.g., [118,119]). Insecticides are present in milkweed plants in and adjacent to agricultural fields in the Midwest [120], and in land use types ranging from agriculture and plant nurseries (where one would expect to find insecticides) to natural and residential areas where pesticides have not been applied. For example, in a 2020 study, all milkweed leaf samples collected from a variety of land use types in the Central Valley of California, contained at least one insecticide [121].

Neonicotinoids became the most widely used insecticides in the world in the early 2000s. Most corn seeds are now treated with neonicotinoids, and their widespread use has caused concern about environmental contamination of water and soil. Their use levels are associated with numbers of bees and butterflies in the UK [122,123] and butterfly species richness in the Upper Midwestern U.S. [111] and lowland California [124]. Unfortunately, understanding ongoing impacts of neonicotinoids is not possible because data on their use have not been collected since 2014 [125]. Concerns about their environmental impacts have resulted in the banning of the four most common and most lethal neonicotinoids in the European Union [126].

Van Deynze et al. [111] documented correlations between insecticide use and monarch numbers, especially neonicotinoids, the use of which, like herbicide-tolerant crops, coincided with the decline in monarch numbers. Recent reviews investigating potential causes of the western monarch decline found that pesticide use is one of the main culprits [6,127]. Thus, the widespread presence of insecticides on the landscape and in milkweed tissue is a major concern, although more research is needed on the impacts of field-level doses.

### 3.4. Other Factors

A number of other factors lead to monarch mortality and could thus affect their numbers from year to year. Monarchs suffer mortality from many natural enemies (predators, parasites, parasitoids, and diseases) during development [103,128,129,130,131] and anything that affects predation rates could be an important population driver. While most experts agree that natural enemies are not as important as habitat and climate in driving monarch numbers [61], it is difficult to assess year to year variation in predation pressure. Predators could become a larger problem if their ranges expand or if they produce more generations under changing climatic conditions, but additional research is needed on the impacts of climate change on predator distribution and abundance.

Consumption of monarchs by invasive and introduced predators could be of particular interest if their impact is greater than that of native predators. Calvert [132,133] showed that invasive fire ants in Texas resulted in almost 100% mortality of monarch eggs and larvae. The invasive multi-colored lady beetle (*Harmonia axyridis*) consumes both monarch eggs and small larvae [134], the introduced paper wasp (*Polistes dominula*) consumes monarch larvae [6,135], and tachinid fly parasitoids, including the introduced *Compsilura concinnata*, can kill a large percentage of monarch larvae [129].

The most well-studied monarch natural enemy is *Ophryocystis elektroschirra* (OE) (summarized by [37]), a protozoan parasite that results in lower fitness and, at high parasite loads, mortality [37,38]. While infection rates of this parasite vary from year to year, it has not been shown to be a major population driver [73]. However, recent research showing that monarchs reared in warmed temperatures were less tolerant of OE infections [136] could indicate that OE impacts will increase under climate warming.

Captive rearing of monarchs may be becoming more common, as people try to help monarchs by helping them avoid predation. In some cases, monarchs are purchased from large-scale breeders. There is concern that purchasing monarchs from breeders has negative impacts due to disease spread, selection for captive conditions that decrease migration propensity, and overall fitness loss, especially when done on a large scale or over multiple generations [137,138,139,140]. However, because it is difficult to quantify the number of monarchs being raised in mass-rearing operations we cannot assess the impact of this practice on monarch numbers.

## 4. How Will Monarchs Fare in the Future?

### 4.1. Ecological Niche Modeling

Researchers use multiple modeling techniques to predict how monarchs will fare in the future. Ecological niche modeling uses observational data to look for non-random associations between monarch occurrences and environmental conditions. In both winter [100] and summer [101,104], the most important factors that determine where monarchs are found are temperature and precipitation. These factors can be projected into the future to determine where the conditions currently used by monarchs will exist. The conditions used by monarchs in the winter will not be available anywhere in Mexico by the middle of the century under climate change scenarios [100], and those used in the summer will occur farther north than they currently do [101,104]. Similar models suggest that the conditions required by the oyamel fir trees in the Mexican wintering sites will move to higher altitudes [91,141] and those required by milkweed species will move north [104].

Zylstra et al. [142] developed a model that incorporated known impacts of weather conditions during the spring and summer on the size of the eastern migratory monarch population. They then used climate projections to predict how monarchs will respond to climate change over the next century. Their models suggested that, overall, monarch numbers will decline, but areas in what is currently the northern part of the summer breeding range will support more monarchs while those in the current southern part of the summer breeding range will have fewer monarchs.

### 4.2. Population Viability Analyses

Population viability analyses (PVAs) can also be used to predict what the future holds for monarchs. These models assess the degree to which populations fluctuate from one year to another and calculate the likelihood that monarch numbers will drop below a level that allows recovery (called an extinction threshold). PVAs are based on the trajectory of population size over time, current population size, the degree to which populations vary from year to year, and the understanding that low numbers make populations vulnerable to catastrophic events. Some PVAs also include predictions of how known drivers might change over time.

Monarch numbers have declined quickly several times in the past (see, for example, declines of over 50% from 1996 to 1997, 2003 to 2004, and 2011 to 2012 in Figure 2), illustrating the possibility of fast declines caused by factors such as severe winter storms in the monarch overwintering area and widespread drought in the spring breeding grounds. Co-occurrence of such events, especially if they happened during a time that the population was already low, could be catastrophic. We do not know the extinction threshold for monarchs (or any extant species), but modelers use input from species experts to estimate this threshold. The higher the estimated extinction threshold, the more likely it is that the population will reach it; likewise, it would be less likely that a population reaches a low extinction threshold.

Multiple PVAs have predicted what many consider unacceptably high extinction risks for the eastern [26,60,61,83] and western [143] migratory populations. Thogmartin [60], using an extinction threshold of 0.25 hectares of occupied winter habitat, estimated that monarchs had a 29% extinction risk in the next decade and a 52% risk in the next 20 years. Semmens et al. [26] and Thogmartin [60] both emphasize the importance of increasing population size as a way of mitigating risk; a population fluctuating around a higher mean size has a lower chance of reaching an extinction threshold. For example, Semmens et al. [26] estimated that a population that occupied 6 hectares of winter habitat would only have extinction risks of 11% and 25% in the next 10 and 20 years, respectively. While PVAs are based on an estimated extinction threshold and predicted future conditions, neither of which can be measured with certainty, they provide the best available estimate of monarchs’ future.

Of the above PVA’s, only three included potential impacts of changing conditions. But given the known impacts of temperature and precipitation, future changes in weather could play a large role in population sustainability. Flockhart et al. [83] incorporated changing conditions at the Mexico overwintering sites. Voorhies et al. [62], using extinction thresholds that ranged from 1 million monarchs (about 0.05 hectare of occupied area at the overwintering sites [144,145]) to 13 million monarchs (about 0.6 hectare) created a comprehensive model that incorporated estimated changes in known drivers of monarch populations: breeding, migrating, and wintering habitat availability; insecticide use; and climate. They considered both changes that would increase the threats to monarchs and conservation practices that could slow declining trends, such as habitat restoration and increased milkweed availability. If conditions remain as they are, Voorhies et al. [62] estimated an extinction risk of 47% in 50 years. Under the best-case scenario conservation action conditions, this risk drops to 36%, and under the worst-case scenario, it increases to 67% for the eastern migratory population. The recent species status assessment [61] includes an update of this analysis and estimates extinction risks of 46 to 67% under best- and worst-case scenarios, respectively.

## 5. Other Perspectives on Monarch Status

### 5.1. Populations Outside of North America

Most of the focus on monarch population status has been on the eastern and western North American migratory populations. However, monarchs exist outside of North America. The existence of these other populations often leads people to say that it is the migratory phenomenon that is at risk and not monarchs themselves. There are some problems with this assertion. While monarchs have been reported in 90 countries, islands, or island groups, they haven’t been observed since 2000 in 21 of these locations [61]. There is little known about most of these populations, including risks they face from the same factors that are affecting North American monarchs. We do know that the populations are all small [8]; about 90% of the monarchs in the world are in North America [61]. Nevertheless, they do provide some redundancy if North American monarchs are extirpated.

### 5.2. Inconsistent Trends Across the Eastern Range

Crossley et al. [68] analyzed NABA counts from 1993 to 2018 that took place in June, July or August to look for site specific trends. They found a slightly positive trend across the entire monarch range, on average. However, they noted declining trends in the U.S. Northeast and the Upper Midwest, the main sources of monarchs migrating to Mexico and where the counts revealed higher abundance. It is unlikely that monarchs in other regions can compensate for losses in their strongholds. Their conclusions were also criticized by Pleasants et al. [89], who argue that summer surveys did not accurately reflect monarch numbers as milkweed was being eradicated from corn and soybean fields; these surveys were conducted in non-agricultural areas and thus reflected a changing proportion of the entire population. Pleasants et al. [89] suggest that winter numbers allow consistent evaluation of the population.

### 5.3. Wintering Outside Traditional Overwintering Sites

Another potential stronghold for monarchs if the migratory phenomenon is lost includes individuals that breed throughout the winter or overwinter without breeding in other locations: along the Gulf Coast, the south Atlantic Coast, and several locations in California [30,32,33,34,46,47,146,147]. We do not know if these populations contribute to the larger population or what makes some monarchs stay in the southeastern U.S. in the winter, but these relatively small populations probably depend on constant influx of migratory individuals [31]. A large proportion of monarchs breeding throughout the winter suffer high loads of OE [33,34,37,146,147], and for this reason Crone and Schultz [148] argued that they are acting as population sinks (but see [6,149]). Finally, cold winter conditions can wipe out monarchs in the southeast and along the Gulf Coast. Despite these uncertainties, coastal overwintering could become more important in the context of declining numbers in Mexico and there is ongoing research on the importance and characteristics of these wintering sites [47].

### 5.4. East-West Exchange

Exchange of individuals between eastern and western populations could provide protective redundancy; if monarchs in one North American population reach low thresholds they could be replenished by individuals from the other population. Monarchs can migrate from the western U.S. to Mexico [39,40], and Brower and Pyle [41] and Dingle et al. [42] suggested that eastern monarchs could replenish the western population. The magnitude of this exchange, and thus the ability of one population to bring the other back from an extinction threshold, is unknown.

### 5.5. Changing Migration Patterns

Another factor that could increase monarchs’ odds of long-term survival is their ability to track changing climatic conditions and move to locations more conducive to their success. Batalden et al. [101] found that monarchs would gain approximately as much breeding habitat as they lost under climate change, assuming that both they and their milkweed host plants would be able to utilize newly available habitat by moving farther north. Lemoine [104] used a similar approach to assess appropriate habitat for both monarchs and milkweed and found an overall increase in suitable summer breeding habitat for eastern monarchs. Of course, the ability of both monarchs and milkweed to move quickly enough is unknown, and it is likely that northern expansion of milkweed will lag behind changing climate conditions [61]. While monarchs are more mobile than milkweed, their northward expansion might also be limited by factors such as the availability of food resources to fuel a longer migration and spring weather conditions that limit breeding in the southern U.S. [67]. Finally, monarchs leaving from their current northern-most breeding areas have lower migratory success [55], so northward movement is likely to increase migratory mortality.

There is some evidence that monarchs are shifting habitat use. Hobson et al. [22], using stable isotope analyses, suggested that the northern reaches of the monarch breeding range are becoming more important. Additionally, monarchs may be choosing different wintering sites, perhaps to find more ideal conditions. For example, most monarchs are usually found in the traditional sites for large colonies (El Rosario, Cerro Pelon, and Sierra Chincua) [150,151,152], but in December 2023, most monarchs were recorded outside of the Monarch Butterfly Biosphere Reserve [153].

### 5.6. Current Stability

Finally, some argue that monarch numbers are no longer declining [59] and are thus not at risk. However, even if this is the case, leveling off around a lower average does present a risk. Weather-driven year-to-year changes could result in monarch numbers declining to an extinction threshold—a number so low that they could not recover [26,60,62]. Of course, for monarchs and any other extant species, we do not know what the extinction threshold is, and it is possible that monarchs will adapt to changing conditions.

## 6. Helping Monarchs: Strategic Habitat Conservation

Increasing the amount and quality of habitat available to monarchs throughout their annual cycle will make them more likely to survive inevitable weather-driven declines [60]. Habitat availability and quality set an upper limit on how many monarchs can be produced, and weather conditions determine if that limit is achieved. While the specifics of recommended habitat conservation action vary with location, the basics are clear. Breeding habitat must contain host and nectar plants to support both larvae and adults. Migratory habitat must contain sufficient nectar resources to fuel the migration and sustain adults during the subsequent winter, as well as safe nighttime stopover sites. When habitat is widespread, it will be easier for females to find host plants for egg laying, making them more likely to lay their full complement of eggs, and adults will be able to find nectar plants as they move during migration and breeding, resulting in longer lives.

Because habitat loss in the breeding range has been identified as a key driver of population size, restoring breeding habitat needs to be a key component of strategic conservation goals [81,90]. This is especially true in the Upper Midwestern U.S., a documented breeding stronghold [154,155] where milkweed has been eradicated in agricultural fields (roughly the middle of the yellow region on Figure 1; [81,90]).

### 6.1. A Regional Perspective

On a regional scale, monarch habitat conservation efforts need to focus on quantity, distribution, and achievability; each of these considerations affect decisions on where habitat conservation should occur.

*Quantity.* Thogmartin et al. [27] and Pleasants [90] calculated that an additional 1.3 to 1.6 billion stems of milkweed are needed on the landscape to achieve a population that would occupy 6 hectares of forest in Mexico, a population size estimated to be stable by Semmens et al. [26]. Adding this much milkweed to the landscape is daunting and can only occur if all potential habitat types are engaged: protected areas, agricultural set-aside land (such as Conservation Reserve Program land), rights-of-ways, urban/suburban land, and land currently in agricultural production [27].

*Distribution.* Monarch conservation efforts spread throughout the entire range will be more effective than focusing on particular regions [13]. Broad habitat availability will make monarchs more resilient because it will be less likely that the entire population will be exposed to adverse conditions at one time. For example, if a drought in the Upper Midwestern U.S. leads to decreased breeding success, monarchs in the Northeastern U.S. may represent a relatively more important proportion of the population, and vice versa. Indeed, the relative importance of different regions changes across years [22,155]. The same reasoning applies to migratory survival. Because monarchs flying north in the spring and early summer cannot anticipate where on the vast landscape habitat will be available, the more that is available to them the more likely they will be to encounter the resources they need to lay their full complement of eggs. Because ideal weather conditions for monarchs are likely to occur farther north in the future, habitat protection, enhancement, and restoration are recommended in northern locations even if they currently host low monarch numbers.

*Achievability.* While adding habitat in the north central and southern parts of the monarch range will lead to a slightly larger increase in population growth than conservation efforts in other parts of the range, combining restoration efforts across all regions will yield population growth with smaller and more achievable investments per region [13]. Thus, habitat across the entire monarch range, essentially everywhere east of the Rocky Mountains except for heavily forested locations, can play a role in maintaining robust, resilient populations.

### 6.2. A Landscape Perspective

Deciding where on the landscape to focus limited conservation resources requires understanding how monarchs move across the landscape—how likely they are to encounter and use habitat patches of different sizes and in different landscape configurations. To gain this understanding, researchers (including hundreds of citizen scientists) estimate monarch abundance across sites in different locations, including per milkweed plant egg and larva density [69,156,157,158], per unit area egg and larva density [156,157,158], and adult butterfly counts [49]. Associating habitat use with habitat characteristics allows us to assign values to habitats with different characteristics. Monarch numbers vary a great deal from year to year, so abundance comparisons across habitats need to take into account the fact that both habitat and the overall size of the population will determine how many monarchs are observed at any given place and time.

*Patch size.* Because larger areas can hold more milkweed and nectar plants, identifying and protecting, enhancing, and restoring large habitat patches should be prioritized. However, even small patches can make important contributions to monarch conservation. Bruce et al. [157] found that patch size did not affect monarch density on grassland sites in Wisconsin that ranged in size from 0.9 to 700 hectares, showing that monarchs find and utilize small patches of habitat. Stenoien et al. [69] found that cultivated gardens, generally small habitat patches, tended to have more monarch eggs per plant than natural areas. Likewise, volunteers in metropolitan Chicago found that approximately 75% of 450 garden patches with milkweed contained monarch eggs [159], especially sites with more milkweed and a higher diversity of blooming plants.

*Roadsides and Other Rights-of-Way.* Kasten et al. [156], in a survey of 1045 randomly selected roadside patches in Minnesota, Wisconsin, and Iowa, found that most patches contained milkweed and monarch eggs and larvae, albeit at lower densities than non-roadside patches being monitored at the same time. Cariveau et al. [158] surveyed 269 Minnesota roadside sites and also found milkweed at most of them. They found that monarch eggs or larvae at 57% of the sites with milkweed, with monarchs more likely at sites with higher milkweed density. While there are concerns about risks to monarchs posed by traffic [160,161] and roadside contaminants such as salt and heavy metals [162,163,164], these risks decrease in areas with lower levels of traffic and Hund et al. [164] found that the amount of salt used on roadsides doesn’t lead to lower fitness. Additionally, well-timed mowing, as can occur on roadsides, provides open habitat and can stimulate milkweed regrowth without resulting in the loss of large numbers of monarch eggs and larvae.

*Proximity to Existing Monarch Habitat.* Monarchs can find even isolated habitat patches. Bruce et al. [157] found that there was a slight negative effect of the amount of grassland within 10 km of a grassland site on monarch egg and larva density. Similarly, Kasten et al. [156] and Cariveau et al. [158] found that per plant egg and larva densities on roadsides were higher when they were not adjacent to grasslands. These studies suggest that isolated patches provide needed islands in otherwise inhospitable habitat. In a modeling study based on what is known about monarch perception and movement patterns, Grant et al. [165] suggested that establishing habitat patches about 50 m apart would facilitate a functionally connected landscape for breeding monarch butterflies, but noted that, because monarchs can move much longer distances, such close placement of habitat patches is probably not necessary. Large, connected patches decrease monarch search time for milkweed and nectar plants, and thus probably increase lifetime fecundity, but their movement patterns across the landscape, especially during migration, mean that protecting and adding habitat in isolated areas can provided needed havens for monarchs on the move. Based on current knowledge, any available habitat can make monarch conservation contributions; isolated patches appear to draw in monarchs from the surrounding area, while connected patches can reduce searching time and lead to higher individual fecundity.

*Pesticide Exposure*. There are different opinions on the importance of situating habitat with respect to sources of insecticide drift, including agricultural fields. We know that monarchs can be harmed by pesticides, and there is a negative association between insecticide use and monarch numbers [11]. As a result, some habitat evaluation guides devalue habitat close to areas treated with insecticides [166,167]. Grant et al. [165] used what is known about insecticide drift, use frequency, and toxicity to analyze risks to monarchs on milkweed in agricultural areas. They concluded that monarch production from areas around untreated fields and from the upwind side of fields would outweigh any reduced production that resulted from mortality caused by insecticide drift.

In conclusion, land managers can be creative about identifying land that could be protected, enhanced, or restored to promote monarch reproduction and adult survival. A large body of research findings suggests that no parcels are too small or too isolated to make a difference.

#### 6.2.1. What to Plant: Milkweed

*Diversity is Good.* Monarchs probably use most of the over 100 milkweed species native to North America and encounter different suites of host plants over the course of their migratory cycle. For example, the most commonly used host plant in the northern breeding range is *Asclepias syriaca* (common milkweed), but there are no records of this species in Texas, Louisiana, Alabama, Georgia, South Carolina, or Florida, and few in any states south of 37° N. Thus, it is most important to select species that are appropriate for the site; female monarchs will find and use them. A diverse selection of appropriate milkweeds will also accommodate within site and between year heterogeneity.

Pocius et al. [168] studied survival of monarchs on nine Midwestern milkweed species and found that monarchs consumed and survived on all species, although survival was lower on *A. hirtella* (tall green milkweed) and *A. sullivantii* (prairie milkweed). To follow up these lab studies, they conducted a three-year evaluation of monarch oviposition on the same nine species planted in arrays at research and demonstration farms in Iowa [169]. Monarchs laid eggs on all species, but *A. incarnata* and *A. syriaca* were preferred when considered across all months and years. Baker and Potter [170] studied monarch oviposition in urban gardens in Lexington, Kentucky and also found that monarchs preferred *A. incarnata* and *A. syriaca*, although plant height appeared to be most important in their study. While these authors recommended prioritizing preferred species in restorations, their recommendations should be used with caution. Different milkweed species are adapted to different growing conditions and will be more robust and thus more attractive to ovipositing females in the habitats that suit them best. For example, *A. exaltata* (poke milkweed), one of the species used in the Iowa study, is a shade-tolerant species that is not found in the open grassland habitats used in that study. Some species are adapted to disturbance and will do well on roadsides and in the garden-like settings used in the Iowa study (e.g., *A. syriaca*) but are often out-competed in pristine prairies with diverse native plant populations. Some species continue growing throughout the year (e.g., *A. verticillata*, whorled milkweed) and thus are likely to be more attractive at the end of the season than species like *A. syriaca* which senesce in late summer.

There is much discussion in the monarch conservation community about *A. curassavica*, tropical milkweed. This species is not native to the U.S., so should not be used in large native plantings. While it is attractive to ovipositing female monarchs, planting it in southern gardens is not recommended for the reasons outlined above (Background on Monarchs). It is unlikely to pose risks to monarchs in the north, but given the abundance and beauty of native species, there is really no reason to use non-native species.

*How much to plant?* Because milkweed seeds are expensive and difficult to harvest and process, land managers are eager for recommendations on how much milkweed seed to include in restoration seed mixes. But making these recommendations is complicated. Even with a targeted restoration density, germination and survival are variable depending on first growth year conditions. Borders and Lee-Mader [171] provide some useful big-picture recommendations. First, milkweed establishment takes time, so it might be 2–3 years until plants are apparent. Second, it is important that milkweed seeds are part of diverse native plant seed mixes. Borders and Lee-Mader recommend including milkweed at rates ranging from 0.1 to 14% and provide a seed count calculator to help determine how much seed will result in a desired seeding rate. In small restorations, it is often easier to plant plugs.

Milkweed density in existing grasslands can provide a baseline for restoration density goals. Lukens et al. [172] measured milkweed density in restored grasslands in Minnesota, Wisconsin, and Iowa, and found an average of 556 plants per acre. Interestingly, *A. syriaca* was present in 60 of 61 restorations, regardless of whether it was included in the seed mix. Whether this would still be true given the loss of so much milkweed on the landscape is unknown; the average age of prairies surveyed by Lukens et al. [172] was eight years, and the sites were surveyed in 2016 and 2017. *Asclepias tuberosa* and *A. incarnata* were more likely to be present if they were planted, and *A. incarnata* was the only species for which density increased with increased seeding rates. Lukens et al. [51] estimated an average of 336 stems per acre in 1227 Integrated Monarch Monitoring Project sites with variable histories across much of the monarch breeding range. These values ranged from a high of 866 stems per acre in roadside sites in the North to a low of 165 stems per acre in randomly selected conservation land in the West. Cariveau et al. [158] found 515 plants per acre in Minnesota roadsides.

While recommendations for how much milkweed to include in a seed mix are complex, there is good evidence that high milkweed densities are beneficial. Kasten et al. [156] and Cariveau et al. [158], studying roadsides, and Bruce et al. [157], studying grasslands, all found higher per plant densities of monarch eggs and larvae when milkweed density was low. However, these teams all found higher egg and larva density per unit area with higher milkweed density. There is no evidence for a saturation density above which more milkweed does not result in more monarch production [157], although few sites at the high end of the density scales were encountered in any of these studies.

The studies summarized above show that eggs are concentrated more densely on fewer plants and spread more thinly across plants when there are more plants. Crowding can result in competition that leads to smaller individuals and lower survival [173,174] as well as higher disease levels [174]. Because females do not appear to respond to the presence of other eggs on a plant by avoiding it for oviposition (personal observations, [173]) the presence of very few plants in a site is likely to lead to crowding.

One conclusion to be drawn from the research cited above is that higher milkweed densities result in higher monarch densities and are likely to prevent detrimental crowding. Additionally, milkweed establishment can result from seeds already in the seed bank or that blow in from other places, but including milkweed in seed mixes, especially species other than *A. syriaca*, is likely to result in their presence in a restoration.

#### 6.2.2. What to Plant: Nectar Plants

Adult monarchs are relatively long-lived and thus require adequate food to lay their full complement of eggs, successfully migrate to wintering sites, and build up enough fat reserves to survive the winter. While it is difficult to link landscape-level nectar availability to population-level impacts on monarchs, there is indirect evidence of its importance. Monarchs acquire lipids as they migrate south in the fall [21,106], resulting in weight gain even during their long flight [20].

During the breeding season, blooming plants attract adult monarchs to sites. Grassland [157] and roadside [158] sites with higher floral richness have higher monarch egg and larva density. Bruce et al. [157] also found that adult monarchs were more abundant in sites with higher floral richness. Thus, diverse nectar plants are important throughout the monarch range to both fuel adult monarchs as they breed, migrate, and overwinter, and to attract females to sites with milkweed.

Because monarch adults are generalists, quantity, diversity, and availability throughout the spring, summer, and fall are probably more important than specific species. A diverse mix of native flowering plants will also benefit other pollinators.

### 6.3. Land Management

As a general rule, land being managed for monarchs doesn’t need to be managed differently. While some management activities (such as fire or mowing during the growing season) might harm individual monarchs, these activities can be timed to avoid high levels of monarch mortality [175]. Most importantly, monarchs will benefit from well-managed habitat: grasslands that are maintained without woody plant encroachment and invasive plants that crowd out milkweed and nectar sources.

There is some evidence that mowing during the growing season makes milkweed more attractive to monarchs [176], although mowing and other management activities should be timed to minimize harm to monarchs whenever possible [175]. Regenerating leaves have higher levels of nitrogen [177] and result in higher survival of young larvae [176]. This kind of disturbance may mimic what occurred in grazed grasslands pre-settlement, although Leone et al. [178] found that heavy grazing had negative impacts on adult monarch density in remnant prairies, and that management with fire resulted in more adult monarchs. *Asclepias syriaca* resprouts particularly well after mowing [176], although drought conditions limit regrowth (personal observations). Other species have not been studied.

## 7. Helping Monarchs: Strategic Monitoring

The Monarch Joint Venture Data and Information Working Group identified several research priorities to better understand how to help monarchs [179]. Many of these priorities can be achieved with relatively easy monitoring. Monitoring will benefit population-wide conservation efforts while at the same time assessing the effectiveness of local conservation actions. Depending on the resources available, prioritizing first milkweed monitoring, then nectar plant monitoring, and finally monarch use of these resources is recommended; this prioritization order begins with both the easiest and the most informative features of the habitat. All are important, but milkweed is often easier to identify and find and is more consistently present throughout the season than nectar plants. Monarch presence and abundance require more time-consuming monitoring and vary a great deal from year to year and throughout the season. The Monarch Joint Venture [180] provides a useful list of existing monitoring programs.

Promising new programs using individual monarch tracking devices are being developed. The Motus Wildlife Tracking System uses tiny (about the weight of a grain of rice), solar-powered radio tags to track monarchs with either hand-held receivers or phone apps [181]. Another promising technology uses tiny sensors, even lighter than the Motus radio tags, to log light intensity and temperature data from the butterfly’s surroundings, which can be remotely read and paired with weather and environmental information using machine learning algorithms [182]. These technologies allow us to track individual butterflies during their migration and even from site to site during the winter. At this point, the cost of individual tags makes their widespread use expensive, but strides in cost-effectiveness, decreasing tag weight, and increasing signal strength means that the technologies hold great promise.

## 8. The Good News

The Eastern North American monarch population is still relatively large and can use a variety of habitats spread out over a large area, especially during the breeding and migratory phases of its annual cycle. This makes the population more resilient to variability and catastrophes than monarch populations elsewhere and other species with smaller ranges. Because female monarchs can lay large numbers of eggs in their lifetimes, the population has the potential to rebound relatively quickly (Figure 2) in years when weather conditions are conducive to successful reproduction and migration. This suggests that raising the habitat-driven limit for monarch numbers could lead to a more stable population. Additionally, the fact that monarchs use different habitat types throughout their annual cycle suggests that they have the means to adapt to changing conditions.

Finally, people care a great deal about monarchs. They are used in educational programming [183], are the focus of many citizen science programs [12] and are the subject of continent-wide conservation efforts [184] that can make a difference [13,27,90,185], benefitting not only monarchs, but the myriad species that share their habitat. This level of attention also provides hope that monarchs will survive ongoing threats to their population.

## Data Availability

No new data were created or analyzed in this study. Data sharing is not applicable to this article.
